# Kidney dysfunction: prevalence and associated risk factors in a community-based study from the North West Province of South Africa

**DOI:** 10.1186/s12882-023-03068-7

**Published:** 2023-01-30

**Authors:** Nonkululeko Hellen Navise, Gontse Gratitude Mokwatsi, Lebo Francina Gafane-Matemane, June Fabian, Leandi Lammertyn

**Affiliations:** 1 Hypertension in Africa Research Team (HART), North-West University, Private Bag x6001, Potchefstroom, South Africa; 2 MRC Unit for Hypertension and Cardiovascular Disease, North-West University, Potchefstroom, South Africa; 3 Wits Donald Gordon Medical Centre, School of Clinical Medicine, Faculty of Health Sciences, University of the Witwatersrand, Johannesburg, South Africa

**Keywords:** Kidney dysfunction, Prevalence, Risk factors, HIV infection, Systolic blood pressure, C-reactive protein

## Abstract

**Background:**

Globally, the World Health Organization ranks chronic kidney disease (CKD) as one of the top 10 causes of mortality. In South Africa, where noncommunicable diseases have become leading causes of mortality, the true population prevalence of CKD is unknown and associated risk factors remain understudied. This study aimed to describe the prevalence of kidney dysfunction and associated risk factors in a community from the North West province of South Africa.

**Methods:**

This cross-sectional study included 1999 participants older than 30 years. Kidney dysfunction was defined as (i) estimated glomerular filtration rate (eGFR) < 90 ml/min/1.73m^2^, or (ii) urine albuminuria-to-creatinine ratio (uACR) ≥ 3.0 mg/mmol, or a combination (i and ii). Risk factors included age, sex, urban/rural locality, body mass index (BMI), blood pressure (BP), lipid profile, haemoglobin A1c (HbA1C), C-reactive protein (CRP), gamma-glutamyl transferase (GGT), tobacco use, and HIV status.

**Results:**

Mean age of participants was 48 (42;56) years, and 655/1999 (33%) had eGFR < 90 ml/min/1.73m^2^ and/or uACR ≥ 3.0 mg/mmol. Compared to those with normal kidney function, participants with eGFR < 90 ml/min/1.73m^2^ and/or uACR ≥ 3.0 mg/mmol were older, female, had higher measures of adiposity, systolic, diastolic, and mean arterial blood pressure, serum lipids and C-reactive protein (CRP) (all p ≤ 0.024). In multiple regression analyses eGFR was associated with systolic BP (β = 0.11) and HIV infection (β = -0.09), and albuminuria was associated with elevated CRP (β = 0.12) and HIV infection (β = 0.11) (all p < 0.026). In both groups (individuals with and without kidney dysfunction respectively), eGFR was associated with age (β = -0.29, β = -0.49), male sex (β = 0.35, β = 0.28), BMI (β = -0.12, β = -0.09), low-density/high-density lipoprotein cholesterol ratio (β = -0.17, β = -0.09) and CRP (β = 0.10, β = 0.09) (all p < 0.005); and uACR was associated with female sex (β = 0.10, β = -0.14), urban locality (β = -0.11, β = -0.08), BMI (β = -0.11, β-0.11), and systolic BP (β = 0.27, β = 0.14) (all p < 0.017).

**Conclusion:**

In this study from the North West province, South Africa, eGFR < 90 ml/min/1.73m^2^ and/or uACR ≥ 3.0 mg/mmol was prevalent and associated with modifiable risk factors. The findings may inform screening strategies for kidney disease prevention, focusing on women, obesity, blood pressure control, dyslipidaemia, identifying and treating inflammation, and HIV diagnosis and treatment.

**Supplementary information:**

The online version contains supplementary material available at 10.1186/s12882-023-03068-7.

## Background

Chronic Kidney Disease (CKD) is an emerging public health challenge with an estimated global prevalence of 13% [1, 2]. In sub-Saharan Africa (SSA), CKD affects approximately 14% of the adult population but varies substantially by region [3]. Despite similar estimated prevalence globally and in SSA, there is disproportionate CKD-associated morbidity and mortality in low- and middle-income settings, including SSA [4–7]. Available data on the burden of CKD in South Africa varies from 2 to 23.9% - in part due to differences in study design and definitions used for CKD [8, 9]. In these South African studies, associated risk for CKD included older age, high body mass index (BMI), sex, cholesterol, diabetes, and hypertension. While the true burden of CKD in SA remains speculative, prevalence is thought to be high because of the rise in noncommunicable diseases (NCD) such as obesity, hypertension, diabetes, and persistent communicable diseases such as HIV infection [10]. Furthermore, many rural communities are undergoing rapid sociodemographic and epidemiological transitions that exacerbate the risk of infectious and NCD [11].

Few population-based studies have been conducted in South Africa to determine CKD prevalence and associated risk factors (modifiable and non-modifiable), which precludes targeted strategies for screening and prevention. The aim of this study was to investigate the prevalence of kidney dysfunction and identify associated risk factors in a mixed urban and rural community from the North West province of South Africa.

## Methods

### Study population and participants

This study formed part of the multinational Prospective Urban and Rural Epidemiological (PURE) study that recruited urban and rural participants 30 years and older from 20 high-, middle-, and low-income countries. The PURE study aimed to examine the impact of social, economic, and demographic factors, and lifestyle behaviour on cardiovascular risk profiles; and determine the incidence of NCD [12]. The PURE study cohort underwent longitudinal follow-up at five and ten years after recruitment with baseline community-based recruitment from 2005. In this study, the baseline cross-sectional data are presented.

Of 4000 individuals approached to participate, 2010 consented adult participants of self-identified black African ethnicity were recruited from two rural (Ganyesa and Tlakgameng) and two urban (Ikageng and Sonderwater extension 7 and 11) areas in the North West province, South Africa. Criteria for inclusion were age, self-identified black African ethnicity, and permanent residence in above-mentioned areas. Participants with missing data on kidney function were excluded (n = 11) and final sample size for this analysis was n = 1999. All participants gave written informed consent before participation and the study was approved by the Health Research Ethics Committee (NWU-00486-20-A1) of the North-West University. All research-related procedures were conducted in accordance with institutional guidelines and the declaration of Helsinki.

### Questionnaires

Questionnaires used in this study were validated for the South African population [13, 14]. With the help of trained field workers, participants completed questionnaires in which they reported information on demographic, health, and lifestyle-related information, including age, sex, alcohol consumption, tobacco use, and medical history.

### Anthropometric measurements

Body height (Invicta Stadiometer, IP 1465, Invicta, London, UK), weight (Electronic scale, Precision Health Scale, A&D Company, Tokyo, Japan), and waist circumferences (WC) (Holtain unstretchable metal tape) were measured using standardised methods according to guidelines of the International Society for the Advancement of Kinanthropometry (ISAK) [15]. We calculated BMI as weight (kg)/height (m^2^).

### Cardiovascular measurements

Clinic brachial blood pressure measurements were determined following standardised guidelines [16] using the Omron HEM-757 device (Omron Healthcare, Kyoto, Japan). Before measurements were taken, participants were required to be seated and resting for 10 min. Two measurements were taken on the left arm with 5-minute intervals between the measurements, with reporting of the second measurement. Pulse pressure (PP) was calculated as the difference between systolic blood pressure (SBP) and diastolic blood pressure (DBP) and mean arterial pressure (MAP) was calculated as DBP added to a third of PP. Hypertension was defined as any of the following (i) SBP ≥ 140 mmHg; (ii) DBP ≥ 90 mmHg; and (iii) current use of anti-hypertension medication.

### Biochemical measurements

Participants were requested to be fasted for approximately 10 h before measurements. Blood samples and a spot urine sample were collected by a registered nurse, stored in ice-filled insulated containers, and transferred to the research laboratory where they were centrifuged, aliquoted, and stored at -20 °C within 2 h of collection. Thereafter, batched samples were transferred to a centralized long-term storage facility (–80 °C biofreezers).

Albumin and creatinine were analysed from serum and spot urine samples using Konelab20i™ auto-analyser (Thermo Fischer Scientific Oy, Vantaa, Finland) and Cobas Integra 400 plus (Roche Diagnostics, Basel, Switzerland), respectively. Serum and urine creatinine was determined by the kinetic colorimetric (Jaffe) assay. Urine albumin was analysed using immunoturbidimetric assay, and uACR was calculated by dividing urine albumin by urine creatinine. Glomerular filtration rate (GFR) was estimated using the Chronic Kidney Disease Epidemiology Collaboration (CKD-EPI) Eq. 2009 for creatinine without adjustment for race [17, 18].

Serum samples were used to determine total cholesterol (TC), high-density lipoprotein cholesterol (HDLC), triglycerides, C-reactive protein (CRP), and gamma-glutamyl transferase (GGT) using Konelab20i™ auto-analyser (Thermo Fischer Scientific, Vantaa, Finland). Low density lipoprotein cholesterol (LDLC) was calculated using the Friedewald formula (LDLC = HDLC – VLDLC where VLDLC = 0.456*TG) [19]. Glucose was analysed in fluoride samples using Vitros DT6011 Chemistry Analyzer (Ortho-Clinical Diagnostics, Rochester, New York, USA). Haemoglobin A1c (HbA1c) was analysed in ethylenediaminetetraacetic acid (EDTA) plasma samples using the D10 Haemoglobin testing system from Bio-Rad (#220 − 0101) that uses ion-exchange high-performance lipid chromatography. Human immunodeficiency Virus (HIV) status was determined according to the South African Department of Health using whole blood for the First Response rapid HIV card test (Premier Medical Corporation Ltd, Daman, India). If the first test result was positive, a second test was performed using an alternative supplier. HIV status was defined as positive if both screening and confirmatory tests were positive (Pareeshak card test (BHA T Biotech, India)).

### Definitions

Using the kidney disease improving global outcomes (KDIGO) 2012 Clinical Practice Guideline for the Evaluation and Management of Chronic Kidney Disease guidelines, the biomarkers used to assess kidney function were estimated GFR (eGFR) and albuminuria (uACR) [20]. In the absence of follow-up testing to confirm abnormalities, we could not fulfil the definition of CKD and instead used the term “kidney dysfunction” rather than “CKD”. For this study, we defined “kidney dysfunction” as eGFR < 90 ml/min/1.73 m^2^ and/or uACR ≥ 3.0 mg/mmol.

### Statistical analyses

We used IBM® SPSS® Statistics, Version 27 software (IBM Corporation, Armonk, New York) for all statistical analyses. The study was able to detect an effect size of 0.33 with a power of 95% using a minimum of 84 participants per eGFR and per uACR group with the significance level set at 0.05 for multiple linear regression with a maximum of 10 covariates. Variables were tested for normality by visual inspection (histograms and q-q plots) as well as skewness and kurtosis coefficients. Continuous data were expressed as median (interquartile range) and categorical data as proportions. The Mann-Whitney U test was used to compare means and proportions between individuals with eGFR ≥ 90 ml/min/1.73m^2^ and uACR < 3.0 mg/mmol; and eGFR < 90 ml/min/1.73m^2^ and/or uACR ≥ 3.0 mg/mmol. Spearman’s rank correlations were used to determine associations between eGFR and uACR (as per the above categories) with lifestyle and cardiometabolic risk factors. We used multiple regression to identify independent associations between various risk factors (age, sex, locality, BMI, SBP, HbA1c, LDLC/HDLC ratio, CRP, GGT, tobacco use, and HIV status) and eGFR and uACR (as per the above categories). Sensitivity analyses were performed for eGFR distributions overall and stratified by sex using (i) the CKD-EPI _(creatinine)_ 2021 equation (no race coefficient) [21]; and (ii) the European Kidney Function Consortium (EKFC) _(creatinine)_ Eq. (22). Sensitivity analyses were also performed to identify independent associations between risk factors and uromodulin. We used z-scores for the continuous variables and actual values for categorical variables.

## Results

Baseline characteristics of the overall study population stratified by kidney function are presented in Table 1. Median age of the study population (n = 1999) was 48 years, 37.2% (743/1999) were men, and 50.2% (1004/1999) were from rural areas. Compared to those with normal kidney function (eGFR ≥ 90 ml/min/1.73m^2^; uACR < 3.0 mg/mmol), individuals with eGFR < 90 ml/min/1.73m^2^ and/or uACR ≥ 3.0 mg/mmol were: older and more likely to be female (both p < 0.001), had higher BMI, WC, blood pressure measures (SBP, DBP and MAP), HbA1c, lipid measures (total cholesterol, LDLC, HDLC and triglycerides), and CRP (all p ≤ 0.024), remained hypertensive despite a higher proportion on antihypertensive medication, and had lower rates of alcohol and tobacco use (all p ≤ 0.023). Characteristics of the study population stratified by sex have been included as Table S1.

**Table 1:** Baseline characteristics of the study population overall, and stratified by kidney function measured as eGFR and uACR

	Overall study population (n = 1999)	(eGFR ≥ 90 ml/min/1.73m^2^ and uACR < 3.0 mg/mmol) (n = 1344)	(eGFR < 90 ml/min/1.73m^2^ and/or uACR ≥ 3.0 mg/mmol) (n = 655)	P*	
Age, years	48 (42;56)	47 (41;55)	51 (44;60)	**< 0.001**	
Sex, men	743/1999 (37.2)	604/1343 (45.0)	139/656 (21.2)	**< 0.001**	
Locality, rural	1004/1999 (50.2)	680/1343 (50.6)	324/656 (49.4)	0.60	
**Body composition**					
BMI, kg/m^2^	24.7 (19.3;28.8)	22.2 (19.0;27.9)	24.5 (20.4;30.2)	**< 0.001**	
WC, cm	77.5 (70.2;87.6)	76.3 (69.9;86.6)	79.6 (70.9;89.8)	**0.001**	
**Cardiovascular measurements**					
SBP, mmHg	129 (116;146)	128 (115;143)	133 (117;152)	**< 0.001**	
DBP, mmHg	87 (78;97)	86 (77;95)	89.0 (80;100)	**< 0.001**	
MAP, mmHg	101 (91;113)	100 (90;111)	104 (93;118)	**< 0.001**	
HT status, n/total (%)	944/1984 (47.6)	587/1334 (56.0)	293/650 (45.1)	**< 0.001**	
**Biochemical measurements**					
eGFR, ml/min/1.73m^2^	101 (87;111)	107 (100;114)	81.3 (70;90)	**< 0.001**	
uACR, mg/mmol	0.59 (0.29;1.46)	0.50 (0.27;1.02)	1.07 (0.36;4.65)	**< 0.001**	
UMOD, µg/ml	36.9 (17.0;66.9)	37.7 (18.0;68.1)	35.3 (14.7;62.7)	**0.035**	
Glucose, mmol/l	4.80 (4.30;5.30)	4.80 (4.30;5.30)	4.80 (4.38;5.30)	0.14	
HbA1c, %	5.50 (5.20;5.80)	5.50 (5.20;5.80)	5.60 (5.30;5.90)	**0.002**	
Diabetes status, n/total (%)	62/1826 (3.2)	36/1216 (3.0)	26/610 (4.3)	0.15	
Total cholesterol, mmol/L	4.82 (4.00;5.87)	4.71 (3.89;5.73)	5.06 (4.22;6.23)	**< 0.001**	
LDLC, mmol/L	2.78 (2.07;3.64)	2.71 (2.02;3.50)	2.92 (2.21;3.93)	**< 0.001**	
HDLC, mmol/L	1.42 (1.06;1.87)	1.38 (1.03;1.86)	1.45 (1.12;1.89)	**< 0.001**	
Triglycerides, mmol/L	1.08 (0.81;1.55)	1.05 (0.80;1.49)	1.14 (0.84;1.66)	**0.022**	
C-reactive protein, mg/L	3.38 (1.01;9.35)	3.04 (0.86;8.74)	4.13 (1.33;12.0)	**0.024**	
GGT, U/L	46.0 (29.7;88.0)	46.4 (29.6;88.0)	45.1 (29.9;85.1)	0.61	
HIV status, n/total (%)	318/1988 (16.0)	202/1336 (15.1)	116/652 (17.8)	0.13	
**Lifestyle factors**					
Alcohol intake, n/total (%)	873/1986 (44.0)	614/1338 (45.9)	259/648 (40.0)	**0.013**	
Tobacco use, n/total (%)	1111/1989 (55.9)	771/1338 (57.6)	340/651 (52.2)	**0.023**	
**Medication use**					
HT medication, n/total (%)	335/1999 (16.8)	186/1343 (13.8)	149/656 (22.7)	**< 0.001**	

Abbreviations: BMI – body mass index; WC – waist circumference; SBP – systolic blood pressure; DBP – diastolic blood pressure; MAP – mean arterial pressure; HT – hypertension; eGFR – estimated glomerular filtration rate; uACR – urine albumin-to-creatinine ratio; HbA1c – haemoglobin A1c; LDLC – low density lipoprotein cholesterol; HDLC – high density lipoprotein; GGT – gamma-glutamyl transferase; HIV – human immunodeficiency virus; HT – hypertension. P value for comparing between kidney function groups. Continuous variables expressed as median (25th to 75th percentile); proportions expressed as number of participants (n/total) and percent (%). GFR estimated using the CKD-EPI _(creatinine)_ 2009 equation without correction for race

Distributions of eGFR and uACR overall, and stratified by sex are detailed in Table 2. For comparison, we performed sensitivity analyses for GFR distributions estimated by the CKD-EPI _(creatinine)_ 2021 (without race) and EKFC _(creatinine)_ equations (Tables S2 and S3).

**Table 2:** Distribution of eGFR and uACR overall, and stratified by sex

eGFR (ml/min/1.73m^2^)	Number (percentages)		
	**Overall**	**Men**	**Women**
≥ 90	1154 (70.8)	548 (90.4)	606 (59.2)
60–89	415 (25.5)	56 (9.2)	359 (35.1)
45–59	47 (2.9)	2 (0.3)	45 (4.4)
30–44	12 (0.7)	-	12 (1.2)
15–29	1 (0.1)	-	1 (0.1)
< 15	0	-	-
uACR (mg/mmol)	Number (percentages)		
	**Overall**	**Men**	**Women**
< 3	1657 (87.0)	619 (87.1)	1038 (87.0)
3–30	234 (12.3)	89 (12.5)	145 (12.2)
> 30	13 (0.7)	3 (0.4)	10 (0.8)

Abbreviations: CKD – chronic kidney disease; eGFR – estimated glomerular filtration rate; uACR – urine albumin-to-creatinine ratio. GFR estimated using the CKD-EPI _(creatinine)_ 2009 equation without correction for race.

Spearman’s rank correlation was used to test for associations between eGFR and uACR and lifestyle and cardiometabolic risk factors to determine the required covariates to use in the multiple regression model (Table S4).

In multivariate regression analyses (Table 3) age, male sex, BMI, SBP, LDLC/HDLC ratio, CRP, and HIV status were factors associated with eGFR in individuals with eGFR < 90 ml/min/1.73m^2^ and/or uACR ≥ 3.0 mg/mmol (all p ≤ 0.026). These associations were also observed in individuals with normal kidney function except for SBP and HIV infection. Risk factors associated with uACR in those with eGFR < 90 ml/min/1.73m^2^ and/or uACR ≥ 3.0 mg/mmol were female sex, urban locality, BMI, SBP, CRP, and HIV infection (all p < 0.017), and the same associations were observed in individuals with normal kidney function with the exception of CRP and HIV infection.

**Table 3:** Multiple-regression analysis for associated risk factors stratified by eGFR and uACR.

	Estimated glomerular filtration rate (mL/min/1.73m^2^)			
	eGFR ≥ 90 ml/min/1.73m^2^ (n = 1344)		eGFR < 90 ml/min/1.73m^2^ (n = 655)	
	**β (95% CI)**	***P***	**β (95% CI)**	***P***
Adjusted R^2^	*0.324; p < 0.001*		*0.272; p < 0.001*	
Age, years	-0.49 (-0.33;-0.26)	**< 0.001**	-0.29 (-0.32;-0.18)	**< 0.001**
Sex, men	0.28 (0.24;0.37)	**< 0.001**	0.35 (0.67;1.04)	**< 0.001**
Locality, rural	0.04 (-0.01;0.10)	0.11	0.03 (-0.09;0.20)	0.42
BMI, kg/m^2^	-0.09 (-0.09;-0.02)	**0.004**	-0.12 (-0.20;-0.04)	**0.005**
SBP, mmHg	0.04 (-0.01;0.05)	0.18	0.11 (0.03;0.17)	**0.005**
HbA1c, %	0.01 (-0.03;0.03)	0.87	-0.01 (-0.08;0.07)	0.85
LDLC/HDLC ratio	-0.09 (-0.08;-0.02)	**< 0.001**	-0.17 (-0.28;-0.12)	**< 0.001**
C-reactive protein, mg/L	0.09 (0.02;0.08)	**0.001**	0.10 (0.03;0.17)	**0.005**
GGT, U/L	0.03 (-0.02;0.06)	0.30	-0.01 (-0.07;0.05)	0.75
HIV infection, (%)	-0.02 (-0.10;0.06)	0.58	-0.09 (-0.42;-0.03)	**0.026**
Tobacco use, (%)	0.01 (-0.05;0.07)	0.73	0.05 (-0.04;0.25)	0.15
	**Urine albumin-to-creatinine ratio (mg/mmol)**			
	uACR < 3.0 mg/mmol (n = 1344)		uACR ≥ 3.0 mg/mmol (n = 655)	
	**β (95% CI)**	***P***	**β (95% CI)**	***P***
Adjusted R^2^	*0.037; p < 0.001*		*0.117; p < 0.001*	
Age, years	0.002 (-0.01;0.01)	0.95	-0.06 (-0.21;0.03)	0.15
Sex, women	-0.14 (-0.06;-0.02)	**< 0.001**	0.10 (0.07;0.73)	**0.017**
Locality, urban	-0.08 (-0.04;-0.01)	**0.007**	-0.11 (-0.61;-0.11)	**0.006**
BMI, kg/m^2^	-0.11 (-0.03;-0.01)	**0.002**	-0.11 (-0.32;-0.04)	**0.013**
SBP, mmHg	0.14 (0.01;0.03)	**< 0.001**	0.27 (0.28;0.53)	**< 0.001**
HbA1c, %	0.003 (-0.01;0.01)	0.92	-0.01 (-0.14;0.12)	0.91
LDLC/HDLC ratio	-0.04 (-0.01;0.002)	0.16	-0.03 (-0.20;0.09)	0.43
C-reactive protein, mg/L	0.05 (-0.002;0.02)	0.12	0.12 (0.07;0.31)	**0.002**
GGT, U/L	0.04 (-0.003;0.02)	0.15	0.01 (-0.08;0.11)	0.80
HIV infection, (%)	0.02 (-0.02;0.03)	0.51	0.11 (0.11;0.73)	**0.011**
Tobacco use, (%)	0.01 (-0.02;0.02)	0.73	0.05 (-0.09;0.41)	0.22

Abbreviations: eGFR – estimated glomerular filtration rate; uACR – urine albumin-to-creatinine ratio; BMI – body mass index; SBP – systolic blood pressure; HbA1c – haemoglobin A1c; LDLC/HDLC ratio – low density lipoprotein cholesterol-to-high density lipoprotein cholesterol ratio; GGT - gamma-glutamyl transferase; HIV – human immunodeficiency virus.

Since the plasma levels of uromodulin have been suggested to be a promising biomarker of kidney function [23], and were identified as significantly lower in the study population with eGFR < 90 ml/min/1.73m^2^ and/or uACR ≥ 3 mg/mmol, we performed a sensitivity analysis to determine whether uromodulin was associated with lifestyle and cardiometabolic factors (Table S5).

## Discussion

In this community-based, cross-sectional study in a mixed urban and rural population from the North West province of South Africa, we observed that risk factors associated with eGFR < 90 ml/min/1.73m^2^ and/or uACR ≥ 3.0 mg/mmol included HIV infection, elevated SBP, and elevated CRP (a non-specific marker of systemic inflammation). Overall, the crude prevalence of albuminuria was 13.0% with no significant sex-related differences in prevalence although levels of albuminuria were significantly higher in women. The crude prevalence of eGFR < 90 ml/min/1.73m^2^ was 29.2% and when compared by eGFR strata, with particular focus on eGFR < 60 ml/min/1.73m^2^, more women than men were affected. Women had higher BMIs and waist circumferences, higher total cholesterol and LDLC, and lower HDLC levels, and more lived in a rural location when compared to men.

The association with HIV, reduced eGFR, and albuminuria is well-described [24, 25]. HIV can directly affect the cellular structure of kidney parenchymal cells, or initiate damage through an immune inflammatory response [26]. The absence of an association with reduced eGFR in our study is notable as several studies have reported a decline in eGFR in HIV-infected populations [27–29]. One potential explanation is that our participants were young with relatively preserved kidney function, and most were ART naïve. The association with albuminuria might have been a consequence of untreated HIV infection, HIV-associated kidney disease, or comorbid infectious disease (most commonly TB). In the absence of confirmatory testing for albuminuria or longitudinal follow-up in this study, whether albuminuria persisted or resolved after initiation of ART remains speculative.

Persistent, low-grade inflammation plays an important role in the pathophysiology of kidney disease [30]. There are many factors that contribute to the inflammatory state, including increased production of proinflammatory cytokines and oxidative stress, acidosis, acute, chronic and recurrent infection [31]. While this study is one of a few in SA to describe the association between albuminuria and markers of inflammation (elevated CRP) this finding is well-described in Asia [32, 33]. One proposed mechanism to explain the association is that elevated CRP reflects the inflammatory milieu, and inflammatory cytokines lead to mesangial cell proliferation, matrix overproduction and increased vascular permeability resulting in albuminuria [34].

Many cardiometabolic risk factors are associated with kidney disease. High blood pressure is closely linked with kidney disease, and it can either be a cause or a consequence of CKD [35]. High blood pressure has been associated with the progression of CKD, the development of CVD and cardiovascular mortality [36–38]. Hypertension is strongly associated with kidney disease in South African populations [10, 39]. There is a high prevalence of hypertension in South Africa [40] and since CKD increases with blood pressure it is not surprising that we found eGFR to associate with SBP in our population.

Obesity is one of the common causes of CKD that lead to the deterioration of structure and function of the kidney [41]. BMI had been shown to be a risk factor associated with CKD in SSA [7]. Our finding confirms that eGFR declines with an increase in obesity [42]. There are, however, conflicting findings on the association of obesity and CKD. Some studies have found counterintuitive associations between obesity and more favourable clinical outcomes in individuals with CKD [43]. The underlying mechanisms of this reverse epidemiology are still unknown. Dyslipidaemia associated with CKD and is characterised by high triglycerides, high LDLC, low HDLC and altered lipoprotein composition [44]. Disturbances in lipoprotein metabolism is seen in individuals with kidney disease and individuals with hypercholesterolemia were found to have a high prevalence of albuminuria and reduced eGFR [45]. We found eGFR to associate with LDLC/HDLC ratio, and according to our knowledge, this is the first study to look at LDLC/HDLC ratio in a South African population.

Disparities in the risk and prevalence of CKD between men and women have been reported. Women tend to have higher prevalence of CKD whereas men are more likely to suffer kidney failure as kidney function declines faster in men[46]. In our study women appeared to have a higher prevalence of CKD and had more advanced stages according to eGFR compared to men. We also found albuminuria to associate with urban locality. Due to rapid urbanisation accompanied by changes in diet, lack of physical activity and increased habits such as smoking and alcohol consumption, it is not surprising that high prevalence of CKD is observed in urban areas [47, 48].

We also looked at the relationship between uromodulin and the risk factors and we found uromodulin to associate with GGT, tobacco use, urban locality, CRP and HIV infection. According to our knowledge these findings have never been described before and future studies are needed to investigate further.

The strength of this study is the large, well-characterised community-based cohort supported by a well-equipped research facility, and inclusion of rural and urban sites in a province of South Africa in which population-based research has not previously been conducted. A major study limitation pertains to the laboratory methodology for creatinine measures. At the time the study was conducted, isotope dilution mass spectrometry (IDMS)-traceable calibrated creatinine assays were not routine laboratory practice. Furthermore, the Jaffe method, rather than the enzymatic, was used for the creatinine assay. In combination, both factors contribute substantial sources of analytic bias in the interpretation of eGFR results and this effect cannot be underestimated. Aside from laboratory-induced bias, non-GFR determinants of creatinine (low muscle mass, low protein ingestion, and possibly, different renal tubular handling of creatinine) are potential contributors to the imprecision of GFR estimating equations in African populations [18] – evident in this study by the discrepant proportions of participants in each eGFR stratum depending on which estimating equation was used. Other limitations of the study included a single measure of eGFR and uACR, without confirmation after three months which precluded confirmation of CKD; the cross-sectional study design which prevented any assessment of causality with lifestyle and cardiometabolic risk factors and measures of kidney function; and limited lifestyle measures data such as diet and physical activity. Most HIV infected individuals were newly diagnosed and ART naive, but for those on ART, no information was collected regarding their treatment and virological control. These factors, and the cross-sectional study design prevented analysis of the effect of ART on the relationship between HIV and kidney function. While elevated CRP is a non-specific marker of inflammation, no further investigations were undertaken to measure additional infectious or inflammatory markers that might have shed light on the association we observed between inflammation with uACR. Lastly, our study was confined to the North West province and may not be generalisable to other ethnic groups in South Africa.

## Conclusion

In this study from urban and rural communities in the North West province, South Africa, the prevalence of eGFR < 90 ml/min/1.73m^2^ and/or uACR ≥ 3.0 mg/mmol was high, and associated with modifiable risk factors. Our findings may inform screening strategies for kidney disease prevention, focusing on women, obesity, systolic blood pressure control, dyslipidaemia, identifying and treating inflammation, and HIV diagnosis and treatment.

## Electronic supplementary material

Below is the link to the electronic supplementary material.

Supplementary Material 1

## Data Availability

The dataset generated and/or analysed during the current study are not publicly available due to contractual agreements but are available from the corresponding author on reasonable request.

## List of abbreviations

BMI: body mass index
CKD: Chronic kidney disease
CKD-EPI: CKD Epidemiology Collaboration
CVD: Cardiovascular disease
CRP: C-reactive protein
DBP: Diastolic blood pressure
eGFR: Estimated glomerular filtration rate
EKFC: European Kidney Function Consortium
GGT: Gamma-glutamyl transferase
HbA1c: Haemoglobin A1c
HDLC: High density lipoprotein cholesterol
HIV: Human immunodeficiency virus
HT: Hypertension
KDIGO: Kidney disease improving global outcomes
LDLC: Low density lipoprotein cholesterol
MAP: Mean arterial pressure
PURE: Prospective Urban and Rural Epidemiological
SBP: Systolic blood pressure
TC: Total cholesterol
uACR: Urine albumin-to-creatinine ratio
UMOD: uromodulin
WC: Waist circumference.

## References

[1] Couser WG, Remuzzi G, Mendis S, Tonelli M (2011). The contribution of chronic kidney disease to the global burden of major noncommunicable diseases. Kidney Int.

[2] Hill NR, Fatoba ST, Oke JL, Hirst JA, O’Callaghan CA, Lasserson DS (2016). Global prevalence of chronic kidney disease - A systematic review and Meta-analysis. PLoS One.

[3] Stanifer JW, Jing B, Tolan S, Helmke N, Mukerjee R, Naicker S (2014). The epidemiology of chronic kidney disease in sub-saharan Africa: a systematic review and meta-analysis. Lancet Glob Health.

[4] Mills KT, Xu Y, Zhang W, Bundy JD, Chen C-S, Kelly TN (2015). A systematic analysis of worldwide population-based data on the global burden of chronic kidney disease in 2010. Kidney Int.

[5] Sumaili EK, Krzesinski J-M, Zinga CV, Cohen EP, Delanaye P, Munyanga SM (2009). Prevalence of chronic kidney disease in Kinshasa: results of a pilot study from the Democratic Republic of Congo. Nephrol Dial Transplant.

[6] Arogundade FA, Barsoum RS (2008). CKD prevention in Sub-Saharan Africa: a call for governmental, nongovernmental, and community support. Am J Kidney Dis.

[7] George JA, Brandenburg J-T, Fabian J, Crowther NJ, Agongo G, Alberts M (2019). Kidney damage and associated risk factors in rural and urban sub-saharan Africa (AWI-Gen): a cross-sectional population study. Lancet Global Health.

[8] Peer N, George J, Lombard C, Steyn K, Levitt N, Kengne A-P (2020). Prevalence, concordance and associations of chronic kidney disease by five estimators in South Africa. BMC Nephrol.

[9] Matsha TE, Yako YY, Rensburg MA, Hassan MS, Kengne AP, Erasmus RT (2013). Chronic kidney diseases in mixed ancestry south african populations: prevalence, determinants and concordance between kidney function estimators. BMC Nephrol.

[10] Moosa M, Van der Walt I, Naicker S, Meyers AJSAMJ (2015). Important causes of chronic kidney disease in South Africa. S Afr Med J.

[11] Kabudula CW, Houle B, Collinson MA, Kahn K, Gómez-Olivé FX, Clark SJ (2017). Progression of the epidemiological transition in a rural south african setting: findings from population surveillance in Agincourt, 1993–2013. BMC Public Health.

[12] Teo K, Chow CK, Vaz M, Rangarajan S, Yusuf S (2009). The prospective Urban Rural Epidemiology (PURE) study: examining the impact of societal influences on chronic noncommunicable diseases in low-, middle-, and high-income countries. Am Heart J.

[13] Keyes CL, Wissing M, Potgieter JP, Temane M, Kruger A, Van Rooy S (2008). Evaluation of the mental health continuum–short form (MHC–SF) in setswana-speaking South Africans. Clin Psychol Psychother.

[14] Goldberg DP, Hillier VF (1979). A scaled version of the General Health Questionnaire. Psychol Med.

[15] ISAK., International Standards for Anthropometric Assessment. ISAK manual, International Society for the Advancement of Kinanthropometry (ISAK): Lower Hutt, New Zealand, 2011.

[16] Muntner P, Shimbo D, Carey RM, Charleston JB, Gaillard T, Misra S (2019). Measurement of blood pressure in humans: A Scientific Statement from the American Heart Association. Hypertension.

[17] Levey AS, Stevens LA (2010). Estimating GFR using the CKD epidemiology collaboration (CKD-EPI) creatinine equation: more accurate GFR estimates, lower CKD prevalence estimates, and better risk predictions. Am J kidney Dis.

[18] Fabian J, Kalyesubula R, Mkandawire J, Hansen CH, Nitsch D, Musenge E (2022). Measurement of kidney function in Malawi, South Africa, and Uganda: a multicentre cohort study. Lancet Glob Health.

[19] Johnson R, McNutt P, MacMahon S, Robson R (1997). Use of the Friedewald formula to estimate LDL-cholesterol in patients with chronic renal failure on dialysis. Clin Chem.

[20] Levin A, Stevens PE, Bilous RW, Coresh J, De Francisco AL, De Jong PE et al. Kidney Disease: Improving Global Outcomes (KDIGO) CKD Work Group. KDIGO 2012 clinical practice guideline for the evaluation and management of chronic kidney disease. Kidney Int Suppl (2011). 2013;3(1):1-150.

[21] Inker LA, Eneanya ND, Coresh J, Tighiouart H, Wang D, Sang Y (2021). New Creatinine- and cystatin C–Based equations to Estimate GFR without Race. N Eng J Med.

[22] Pottel H, Björk J, Courbebaisse M, Couzi L, Ebert N, Eriksen BO (2021). Development and validation of a modified full age Spectrum Creatinine-Based equation to Estimate glomerular filtration rate: a cross-sectional analysis of Pooled Data. Ann Intern Med.

[23] Steubl D, Block M, Herbst V, Nockher WA, Schlumberger W, Satanovskij R (2016). Plasma uromodulin correlates with kidney function and identifies early stages in chronic kidney Disease patients. Med (Baltim).

[24] Kim PS, Woods C, Dutcher L, Georgoff P, Rosenberg A, Mican JAM (2011). Increased prevalence of albuminuria in HIV-infected adults with diabetes. PLoS ONE.

[25] Wensink GE, Schoffelen AF, Tempelman HA, Rookmaaker MB, Hoepelman AIM, Barth RE (2015). Albuminuria is associated with traditional cardiovascular risk factors and viral load in HIV-infected patients in rural South Africa. PLoS ONE.

[26] Alfano G, Cappelli G, Fontana F, Di Lullo L, Di Iorio B, Bellasi A (2019). Kidney disease in HIV infection. J Clin Med.

[27] Alves TP, Hulgan T, Wu P, Sterling TR, Stinnette SE, Rebeiro PF (2010). Race, kidney disease progression, and mortality risk in HIV-infected persons. Clin J Am Soc Nephrol.

[28] Jotwani V, Li Y, Grunfeld C, Choi AI, Shlipak MG (2012). Risk factors for ESRD in HIV-infected individuals: traditional and HIV-related factors. Am J Kidney Dis.

[29] Campbell L, Ibrahim F, Fisher M, Holt S, Hendry B, Post F (2009). Spectrum of chronic kidney disease in HIV-infected patients. HIV Med.

[30] Mihai S, Codrici E, Popescu ID, Enciu A-M, Albulescu L, Necula LG (2018). Inflammation-related mechanisms in chronic kidney disease prediction, progression, and outcome. J Immunol Res.

[31] Akchurin M, Kaskel F (2015). Update on inflammation in chronic kidney disease. Blood Purif.

[32] Zambrano-Galvan G, Rodríguez-Morán M, Simental-Mendía LE, Lazalde B, Reyes-Romero MA, Guerrero-Romero F (2011). C-reactive protein is directly Associated with urinary albumin-to-creatinine ratio. Arch Med Res.

[33] Sabanayagam C, Lee J, Shankar A, Lim SC, Wong TY, Tai ES (2010). C-reactive protein and microalbuminuria in a multi-ethnic asian population. Nephrol Dial Transplant.

[34] Imig JD, Ryan MJ (2013). Immune and inflammatory role in renal disease. Compr Physiol.

[35] Levin NW, Kotanko P, Eckardt K-U, Kasiske BL, Chazot C, Cheung AK et al. Blood pressure in chronic kidney disease stage 5D-report from a Kidney Disease: Improving Global Outcomes controversies conference. Kidney Int. 2010;77(4):273 – 84. 10.1038/ki.2009.469 20016467

[36] Lee JY, Park JT, Joo YS, Lee C, Yun H-R, Yoo T-H (2021). Association of blood pressure with the progression of CKD: findings from KNOW-CKD Study. Am J of Kidney Dis.

[37] Xie X, Atkins E, Lv J, Bennett A, Neal B, Ninomiya T (2016). Effects of intensive blood pressure lowering on cardiovascular and renal outcomes: updated systematic review and meta-analysis. Lancet.

[38] Wu Z, Jin C, Vaidya A, Jin W, Huang Z, Wu S (2016). Longitudinal patterns of blood pressure, incident cardiovascular events, and all-cause mortality in normotensive diabetic people. Hypertension.

[39] Matsha TE, Kengne AP, Masconi KL, Yako YY, Erasmus R (2015). APOL1 genetic variants, chronic kidney diseases and hypertension in mixed ancestry South Africans. BMC Genet.

[40] Ware LJ, Chidumwa G, Charlton K, Schutte AE, Kowal P (2019). Predictors of hypertension awareness, treatment and control in South Africa: results from the WHO-SAGE population survey (Wave 2). J Hum Hypertens.

[41] Garland JS (2014). Elevated body mass index as a risk factor for chronic kidney disease: current perspectives. Diabetes Metab Syndr Obes.

[42] Adeniyi AB, Laurence CE, Volmink JA, Davids MR (2017). Prevalence of chronic kidney disease and association with cardiovascular risk factors among teachers in Cape Town, South Africa. Clin Kidney J.

[43] Kalantar-Zadeh K, Rhee CM, Chou J, Ahmadi SF, Park J, Chen JL (2017). The obesity Paradox in kidney disease: how to reconcile it with obesity management. Kidney Int Rep.

[44] Cases A, Coll E (2005). Dyslipidemia and the progression of renal disease in chronic renal failure patients. Kidney Int.

[45] Liu D-w, Jia W, Liu Z-s, Pei W, Cheng G-Y, Shi X-Z (2013). Association between dyslipidemia and chronic kidney disease: a cross-sectional study in the middle-aged and elderly chinese population. Chin Med J.

[46] Goldberg I, Krause I (2016). The role of gender in chronic kidney disease. Eur Med J.

[47] Adjei DN, Stronks K, Adu D, Beune E, Meeks K, Smeeth L (2018). Chronic kidney disease burden among african migrants in three european countries and in urban and rural Ghana: the RODAM cross-sectional study. Nephrol Dial Transplant.

[48] Stanifer JW, Maro V, Egger J, Karia F, Thielman N, Turner EL (2015). The epidemiology of chronic kidney disease in Northern Tanzania: a population-based survey. PLoS ONE.

