# Supplementary material for: Kidney dysfunction: prevalence and associated risk factors in a community-based study from the North West Province of South Africa
Source: BMC Nephrol. 2023 Jan 30;24:23. doi: 10.1186/s12882-023-03068-7 (PMC9887915; doi:10.1186/s12882-023-03068-7)
Supplement: Supplementary file 1 — Supplementary Material 1 [file 12882_2023_3068_MOESM1_ESM.docx]

**Supplementary material**

| **Table S1.** Baseline characteristics overall, and stratified by sex | | | | |  |
| --- | --- | --- | --- | --- | --- |
|  | Total group  (n=1999) | Men  (n=743) | Women  (n=1256) | P* |  |
| Age, years | 48.0 (42.0;56.0) | 49.0 (42.0;57.0) | 48.0 (42.0;56.0) | 0.20 |  |
| Locality, rural | 1004/1999 (50.2) | 347/743 (46.7) | 657/1256 (52.3) | **0.015** |  |
| **Body composition** | | | | |  |
| BMI, kg/m^2^ | 24.7 (19.3;28.8) | 19.8 (18.1;22.4) | 26.0 (21.6;31.8) | **<0.001** |  |
| WC, cm | 77.5 (70.2;87.6) | 74.4 (69.9;81.2) | 81.0 (70.6;91.2) | **<0.001** |  |
| **Cardiovascular measurements** | | | | |  |
| SBP, mmHg | 129 (116;146) | 132 (118;148) | 127 (114;145) | **<0.001** |  |
| DBP, mmHg | 87.0 (78.0;97.0) | 86.0 (77.0;96.0) | 87.5 (78.0;97.0) | **0.018** |  |
| MAP, mmHg | 101 (91.0;113) | 101 (91.0;112) | 101 (91.0;113) | 0.93 |  |
| HT status, n/total (%) | 944/1984 (47.6) | 348/738 (47.2) | 596/1246 (47.8) | 0.77 |  |
| **Biochemical measurements** | | | | |  |
| eGFR, ml/min/1.73m^2^ | 101 (86.7;111) | 109 (99.6;118) | 95.3 (79.6;107) | **<0.001** |  |
| uACR, mg/mmol | 0.59 (0.29;1.46) | 0.53 (0.27;1.45) | 0.61 (0.30;1.47) | 0.59 |  |
| UMOD, µg/ml | 36.9 (17.0;66.9) | 38.6 (19.2;71.0) | 36.1 (16.4;65.1) | 0.45 |  |
| Glucose, mmol/L | 4.80 (4.30;5.30) | 4.70 (4.30;5.20) | 4.80 (4.30;5.30) | **0.005** |  |
| HbA1c, % | 5.50 (5.20;5.80) | 5.50 (5.20;5.70) | 5.60 (5.30;5.90) | **<0.001** |  |
| Diabetes status, n/total (%) | 62/1826 (3.2) | 18.0/699 (2.6) | 44.0/1127 (3.9) | 0.13 |  |
| Total cholesterol, mmol/L | 4.82 (4.00;5.87) | 4.61 (3.82;5.58) | 4.94 (4.14;6.03) | **<0.001** |  |
| LDLC, mmol/L | 2.78 (2.07;3.64) | 2.56 (1.86;3.35) | 2.90 (2.23;3.77) | **<0.001** |  |
| HDLC, mmol/L | 1.42 (1.06;1.87) | 1.48 (1.07;1.98) | 1.37 (1.06;1.81) | **0.001** |  |
| Triglycerides, mmol/L | 1.08 (0.81;1.55) | 0.98 (0.77;1.41) | 1.14 (0.85;1.65) | **0.001** |  |
| C-reactive protein, mg/L | 3.38 (1.01;9.35) | 2.50 (0.76;8.01) | 3.77 (1.27;10.4) | 0.58 |  |
| GGT, U/L | 46.0 (29.7;88.0) | 58.3 (36.8;123) | 40.3 (26.6;70.9) | **<0.001** | |
| HIV status, n/total (%) | 318/1988 (16.0) | 116/741 (15.7) | 202/1247 (16.2) | 0.75 |  |
| **Lifestyle factors** | | | | |  |
| Alcohol intake, n/total (%) | 873/1986 (44.0) | 489/739 (66.2) | 384/1247 (30.8) | **<0.001** |  |
| Tobacco use, n/total (%) | 1111/1989 (55.9) | 493/739 (66.7) | 618/1250 (49.4) | **<0.001** |  |
| **Medication use** | | | | |  |
| HT medication, n/total (%) | 335/1999 (16.8) | 78.0/743 (10.5) | 257/1256 (20.5) | **<0.001** |  |
| Abbreviations: BMI – body mass index; WC – waist circumference; SBP – systolic blood pressure; DBP – diastolic blood pressure; MAP – mean arterial pressure; HT – hypertension; eGFR – estimated glomerular filtration rate; uACR – urine albumin-to-creatinine ratio; HbA1c – haemoglobin A1c; LDLC – low density lipoprotein cholesterol; HDLC – high density lipoprotein; GGT – gamma-glutamyl transferase; HIV – human immunodeficiency virus; HT – hypertension. P value for between sex comparison. Continuous variables expressed as median (25th to 75th percentile); proportions expressed as number of participants (n/total) and percent (%). GFR estimated using the CKD-EPI _(creatinine)_ 2009 equation without correction for race. | | | | |  |

| **Table S2.** Distributions of eGFR overall and stratified by sex using the CKD-EPI _(creatinine)_ 2021 equation without race (21).  Population reference for creatinine: men (80µmol/L/0.9mg/dl); women (62µmol/L/0.7mg/dl) | | | |
| --- | --- | --- | --- |
| eGFR (ml/min/1.73m^2^) | Number (percent) | | |
|  | **Overall** | **Men** | **Women** |
| >90 | 1256 (77.1) | 576 (95.0) | 680 (66.5) |
| 60-89 | 334 (20.5) | 29 (4.8) | 305 (29.8) |
| 45-59 | 30 (1.8) | 1 (0.2) | 29 (2.8) |
| 30-44 | 8 (0.5) | 0 | 8 (0.8) |
| 15-29 | 0 | 0 | 0 |
| <15 | 0 | 0 | 0 |

| **Table S3.** Distributions of eGFR overall and stratified by sex using the EKFC _(creatinine)_ equation (22).  EKFC population reference for creatinine: men (80µmol/L/0.9mg/dl); women (62µmol/L/0.7mg/dl) | | | |
| --- | --- | --- | --- |
| eGFR (ml/min/1.73m^2^) | Number (percent) | | |
|  | **Overall** | **Men** | **Women** |
| >90 | 1047 (64.3) | 507 (83.7) | 540 (52.8) |
| 60-89 | 515 (31.6) | 96 (15.8) | 419 (41.0) |
| 45-59 | 53 (3.3) | 3 (0.5) | 50 (4.9) |
| 30-44 | 12 (0.7) | 0 | 12 (1.2) |
| 15-29 | 1 (0.1) | 0 | 1 (0.1) |
| <15 | 0 | 0 | 0 |
| Study sample population reference for creatinine: men (65µL/0.74 mg/dl); women (66µL 0.75 mg/dl) | | | |
| eGFR (ml/min/1.73m^2^) | Number (percent) | |  |
|  | **Overall** | **Men** | **Women** |
| ≥90 | 1132 (69.5) | 370 (61.1) | 762 (60.7) |
| 60-89 | 465 (28.5) | 221 (36.5) | 244 (23.9) |
| 45-59 | 26 (1.6) | 14 (2.3) | 12 (1.2) |
| 30-44 | 6 (0.4) | 1 (0.2) | 5 (0.5) |
| 15-29 | 0 | 0 | 0 |
| <15 | 0 | 0 | 0 |
|  | | | |

| **Table S4.** Spearman’s correlation testing associations between eGFR and uACR, and lifestyle and cardiometabolic risk factors | | | | | | | | |
| --- | --- | --- | --- | --- | --- | --- | --- | --- |
|  | **Estimated glomerular filtration rate (ml/min/1.73m^2^)** | | | | **Urine albumin-to-creatinine ratio (mg/mmol)** | | | |
|  | ≥90ml/min/1.73m^2^ | | <90ml/min/1.73m^2^ | | <3.0 mg/mmol | | ≥3.0 mg/mmol | |
|  | ***r_2_*** | ***p*** | ***r_2_*** | ***p*** | ***r_2_*** | ***p*** | ***r_2_*** | ***p*** |
| Age, years | **-0.47** | **<0.001** | **-0.15** | **<0.001** | **0.08** | **0.008** | 0.03 | 0.39 |
| Sex, men | **0.28** | **<0.001** | **0.36** | **<0.001** | **-0.08** | **0.003** | **0.26** | **<0.001** |
| Locality, rural | 0.06 | 0.081 | 0.07 | 0.081 | **-0.08** | **0.004** | **-0.18** | **<0.001** |
| BMI, kg/m^2^ | **-0.24** | **<0.001** | **-0.31** | **<0.001** | **-0.06** | **0.028** | **-0.26** | **<0.001** |
| WC, cm | **-0.20** | **<0.001** | **-0.21** | **<0.001** | **-0.07** | **0.021** | **-0.19** | **<0.001** |
| SBP, mmHg | **-0.09** | **0.003** | 0.03 | 0.42 | **0.11** | **<0.001** | **0.27** | **<0.001** |
| DBP, mmHg | **-0.10** | **0.001** | 0.07 | 0.072 | **0.12** | **<0.001** | **0.30** | **<0.001** |
| HT status, n/total (%) | **-0.09** | **0.005** | 0.06 | 0.17 | **0.14** | **<0.001** | **0.21** | **<0.001** |
| UMOD, (µg/ml) | -0.03 | 0.41 | **-0.09** | **0.039** | **-0.44** | **<0.001** | **-0.34** | **<0.001** |
| Glucose, mmol/L | **-0.12** | **<0.001** | 0.01 | 0.84 | -0.01 | 0.74 | 0.02 | 0.60 |
| HbA1c, % | **-0.13** | **<0.001** | **-0.15** | **<0.001** | -0.02 | 0.49 | **-0.09** | **0.021** |
| Diabetes status, n/total (%) | 0.01 | 0.84 | -0.02 | 0.57 | 0.02 | 0.55 | 0.02 | 0.71 |
| Total cholesterol, mmol/L | **-0.13** | **<0.001** | **-0.12** | **0.003** | 0.02 | 0.57 | -0.07 | 0.10 |
| LDLC, mmol/L | **-0.16** | **<0.001** | **-0.22** | **<0.001** | -0.04 | 0.14 | **-0.12** | **0.003** |
| HDLC, mmol/L | 0.05 | 0.12 | **0.15** | **<0.001** | **0.09** | **0.002** | 0.07 | 0.070 |
| Triglycerides, mmol/L | **-0.18** | **<0.001** | -0.02 | 0.55 | 0.02 | 0.60 | 0.02 | 0.56 |
| C-reactive protein, mg/L | -0.03 | 0.33 | -0.03 | 0.49 | 0.04 | 0.19 | 0.04 | 0.33 |
| GGT, U/L | **0.11** | **0.001** | **0.22** | **<0.001** | **0.07** | **0.023** | **0.24** | **<0.001** |
| HIV status, n/total (%) | **0.09** | **0.003** | -0.03 | 0.41 | 0.01 | 0.68 | 0.07 | 0.098 |
| Alcohol intake, n/total (%) | **0.16** | **<0.001** | **0.21** | **<0.001** | 0.05 | 0.067 | **0.26** | **<0.001** |
| Tobacco use, n/total (%) | **0.09** | **0.003** | **0.12** | **0.003** | 0.03 | 0.27 | **0.12** | **0.002** |
| HT medication, n/total (%) | **-0.16** | **<0.001** | **-0.14** | **<0.001** | **0.07** | **0.017** | 0.01 | 0.79 |
| Abbreviations: eGFR – estimated glomerular filtration rate; uACR – urine albumin-to-creatinine ratio; BMI – body mass index; WC – waist circumference; SBP – systolic blood pressure; DBP – diastolic blood pressure; MAP – mean arterial pressure; HT – hypertension; UMOD – uromodulin; HbA1c – haemoglobin A1c; LDLC – low density lipoprotein cholesterol; HDLC – high density lipoprotein; GGT – gamma-glutamyl transferase; HIV – human immunodeficiency virus; HT – hypertension. GFR estimated using the CKD-EPI _(creatinine)_ 2009 equation without correction for race. | | | | | | | | |

| **Table S5.** Multiple-regression analysis for uromodulin and associated risk factors stratified by kidney function (eGFR and uACR) | | | | |
| --- | --- | --- | --- | --- |
|  | **Uromodulin (µg/ml)** | | | |
|  | eGFR ≥90 ml/min/1.73m^2^ and uACR <3.0 mg/mmol  (n=1344) | | eGFR <90 ml/min/1.73m^2^; and/or uACR ≥3.0 mg/mmol  (n=655) | |
|  | **β (95% CI)** | ***P*** | **β (95% CI)** | ***P*** |
| Adjusted R^2^ | *0.026; p<0.001* | | *0.055; p<0.001* | |
| Age, years | 0.02 (-0.04;0.09) | 0.45 | 0.02 (-0.07;0.11) | 0.65 |
| Sex, men | 0.05 (-0.07;0.18) | 0.42 | -0.01 (-0.24;0.21) | 0.92 |
| Locality, rural | -0.18 (-0.29;-0.07) | **0.002** | -0.24 (-0.41;-0.06) | **0.007** |
| BMI, kg/m^2^ | 0.01 (-0.06;0.08) | 0.84 | 0.04 (-0.05;0.14) | 0.35 |
| SBP, mmHg | -0.04 (-0.10;0.02) | 0.22 | -0.06 (-0.15;0.02) | 0.15 |
| HbA1c, % | -0.04 (-0.09;0.02) | 0.24 | 0.01 (-0.08;0.10) | 0.87 |
| LDLC/HDLC ratio | 0.04 (-0.02;0.09) | 0.19 | 0.01 (-0.08;0.11) | 0.77 |
| C-reactive protein, mg/L | 0.12 (0.07;0.18) | **<0.001** | 0.15 (0.07;0.24) | **<0.001** |
| GGT, U/L | 0.01 (-0.06;0.08) | 0.76 | 0.10 (0.02;0.15) | **0.014** |
| HIV infection, (%) | 0.18 (0.03;0.34) | **0.023** | -0.12 (-0.36;0.11) | 0.30 |
| Tobacco use, (%) | 0.02 (-0.10;0.14) | 0.74 | -0.24 (-0.41;-0.06) | **0.008** |
| Abbreviations: BMI – body mass index; SBP – systolic blood pressure; HbA1c – haemoglobin A1c; LDLC/HDLC ratio – low density lipoprotein cholesterol – to – high density lipoprotein cholesterol ratio; GGT - gamma-glutamyl transferase; HIV – human immunodeficiency virus. | | | | |
